# Utilizing Xanthan Gum Coatings as Probiotic Bacteria Carriers to Enhance Postharvest Quality and Antioxidants in Fresh-Cut Cantaloupe and Honeydew (*Cucumis melo* L.) Melons

**DOI:** 10.3390/foods13060940

**Published:** 2024-03-20

**Authors:** Tshudufhadzo Chikhala, Faith Seke, Retha M. Slabbert, Yasmina Sultanbawa, Dharini Sivakumar

**Affiliations:** 1 Department of Horticulture, Tshwane University of Technology, Pretoria West 0001, South Africa; chudufhadzo@gmail.com (T.C.); slabbertmm@tut.ac.za (R.M.S.); 2 Phytochemical Food Network Group, Department of Crop Sciences, Tshwane University of Technology, Pretoria West 0001, South Africa; sekef@tut.ac.za; 3 Australian Research Council Industrial Transformation Training Centre for Uniquely Australian Foods, Queensland Alliance for Agriculture and Food Innovation, Centre for Food Science and Nutrition, The University of Queensland, St Lucia, QLD 4069, Australia; y.sultanbawa@uq.edu.au

**Keywords:** edible coating, bioactive compounds, antioxidant properties, lactobacillus strains, color properties, sensory properties

## Abstract

Due to spoilage microflora and browning, minimally processed fresh-cut fruits have a short shelf life, and over the years, studies have shown the potential of using edible coatings to extend the shelf life and improve the safety of fresh-cut fruits. Recently, there has been a rise in research on the incorporation of probiotics in edible coatings due to the bespoke health and biopreservation benefits they impart. Therefore, in this study, lactobacillus strains (*Lactiplantibacillus plantarum* 75 and *Bifidobacterium longum*) were incorporated into a xanthan edible coating to enhance color retention, sensory properties, antioxidant retention (ascorbic acid, carotenoids, total phenols), and antioxidant activity (FRAP antioxidant power, ABTS scavenger activity) of fresh-cut cantaloupes and honeydew *cucumis melo* L. melons during cold storage at 5 C and 85% RH for five days. The edible coating was prepared by mixing 0.5% xanthan gum, 1% glycerol, and 2% citric acid solution with *L. plantarum 75* (LAB 75) and *Bifidobacterium longum* bacteria separately, and the final lab count for each strain was made to be 8.0 log CFU/mL. Stable probiotic coatings with ζ-potential of between −39.7 and −51.4 mV and a PdI of 1 were developed, and the incorporation of the probiotic bacteria into the coating was justified using FTIR analysis. The probiotic coatings showed a typical pseudoplastic behavior, in which the viscosity curves fall as the shear rate increases. Thermal stability analysis showed a continuous and multi-step weight reduction in this work, illustrating how the edible coating components interact. The survival of both Lactobacillus strains was recorded on day 5. Both freshly cut melons coated with xanthan and loaded with Lactobacillus strains retained a sufficient quantity of probiotics at the end of storage, while *L. plantarum 75* (7 log CFU/g for cantaloupe and 8 log CFU/g for honeydew) retained the highest viability compared to *B. longum* (6 log CFU/g for cantaloupe and 7 log CFU/g for honeydew). In comparison to the coated and uncoated control samples, the inclusion of *L. plantarum 75* in xanthan coatings significantly retained the color properties, pigments (total chlorophyll and carotenoids), ascorbic acid, total phenols, and antioxidant activity (FRAP, DPPH, and ABTS). The overall acceptability of fresh cuts of cantaloupe and honeydew melons coated with xanthan gum loaded with *L. plantarum 75* was higher than that of other treatments. Thus, xanthan gum loaded with *L. plantarum 75* coating is most suitable for reducing postharvest losses in fresh cuts of honeydew melons and cantaloupe, which will help preserve antioxidant and bioactive properties. The xanthan gum loaded with *L. plantarum 75* coatings exhibited the highest preservation impact; therefore, it can be recommended for the fresh-cut industry.

## 1. Introduction

Fresh or minimally processed fruits and vegetables are becoming more popular as consumers seek out healthy, chemical-free, high-quality food. Fresh fruits and vegetables have become more popular due to their nutritional value, general acceptance, and affiliation with various health benefits. Fresh-cut melon (*Cucumis melo* L.) is a popular fruit choice among consumers due to its desired sensory and nutritional qualities. Cantaloupes and honeydew melons are good sources of vitamin C, beta-carotene, and potassium and are low in fat and sodium [1]. Cantaloupe has a higher beta-carotene content than honeydew cultivars [2]. 

Fresh-cut melons meet consumer demand as a fast-prepared, convenient, and healthy food [3]. Minimally processed fresh-cut fruits have a limited shelf life because of the deteriorative activities of spoilage microflora and physiological processes [4], which could negatively impact the sensory quality [5]. Some visible effects of quality depreciation in fresh-cut produce include discoloration, increased oxidative browning on cut surfaces, flaccidity resulting from water loss, and decreased nutritional quality. It is important to develop alternative methods for enhancing the quality and safety of minimally processed or fresh-cut products. Accordingly, Iglesias et al. [6] and Zudaire et al. [7] assert that probiotics are an innovative technology for bioprotecting fresh fruits and vegetables minimally processed after harvest. Beneficial microorganisms can aid in the prevention of foodborne diseases by producing specific metabolites that can alter the pH, moisture, and nutrient content of fruits and vegetables [8]. Furthermore, the minerals, vitamins, antioxidants, and fiber content of minimally processed fruits and vegetables make them excellent substrates for probiotic cultures, Soccol et al. [9]. Therefore, incorporating probiotics and vegetables can help extend the shelf life of fruits without the need for chemical antimicrobials. According to the Food and Agriculture Organisation of the United Nations and the World Health Organization, probiotics contain live bacteria that offer health benefits to consumers in the appropriate amounts. *Lactobacillus* and *Bifidobacterium* are the most widely used probiotics. Lactic acid bacteria (LAB) are generally recognized as safe (GRAS) according to the United States Food and Drug Administration [10]. Currently, consumers are shifting their habits towards limiting their consumption of products derived from animals, such as those without lactose and cholesterol. The probiotics market is projected to grow by 14.0% from 2023 to 2030, reaching USD 220.14 billion. 

Nonetheless, fruits and vegetables may undergo undesirable effects when probiotics are directly added to their surfaces, compromising their consumer appeal and lowering the ability of probiotics to survive in storage [11]. It has been suggested that edible coatings can overcome the limitations associated with fresh and minimally processed fruit and vegetables compromised probiotic activity [12]. Edible coatings enhance safety, protect the sensory and nutritional qualities of coated products, and serve as probiotic carriers [11]. An edible matrix must facilitate easy delivery methods that are more affordable to ensure probiotic survival [12]. For consumers, *Bifidobacterium* and *Lactiplantibacillus plantarum* have potential health benefits and biological functions, including the production of short-chain fatty acids, such as lactic and acetic acids. These acids lower the pH, improve calcium and magnesium availability, and inhibit potentially pathogenic bacteria in the gastrointestinal tract [13]. *Bifidobacterium longum* is an essential probiotic bacterium that thrives in the human gastrointestinal tract. Currently, it is found in dietary supplements, food, health products, and drugs to balance intestinal bacteria, boost immunity, increase lipid metabolism, ease constipation, and provide antioxidant properties [14]. Moreover, in recent years, non-dairy probiotic foods have become increasingly popular among lactose-intolerant consumers. In addition to functional foods, probiotic functional foods are also growing in popularity among consumers [8,15,16]. 

Furthermore, the use of edible coatings made from various biopolymer materials (such as alginate, pectins, gellan, chitosan, caseinate, and k-carrageenan) may enhance the nutritional and sensory properties of fruits [17,18,19]. These coatings extend the shelf life of fruits while reducing water loss and fungal growth [20]. Additionally, they preserve the food products’ quality characteristics, appearance, and shelf life because of their promising barrier qualities that prevent the transfer of moisture and gases, oxidize lipids, regulate enzyme activity, and prevent microbiological decomposition [21]. Tapia et al. [22] investigated the impact of alginate and gellan-based edible coating for probiotic coatings on fresh cuts of apples and papaya, focusing on the techno-functional properties of the coatings and enumerating the viability of *Bifidobacterium* in the coatings. Edible functionalized coating systems with probiotics are becoming increasingly popular. Carboxymethyl cellulose (CMC) and *Lactobacillus plantarum* applied to strawberries reduced weight loss and decay and slowed the deterioration of ascorbic acid and phenolic compounds during storage time [23]. CMC (1% *w*/*v*) and Zein powder (from maize, Sigma-Aldrich, 5% *w*/*v*) bilayer coating combined with *Lactobacillus plantarum* 299v improved the shelf life and safety quality of fresh cut apple slices [24]. Sparanza et al. [25] reported that alginate coating loaded with *Lactobacillus plantarum* displayed superior performance without any adverse effects on probiotic viability, and treatments involving probiotic coatings also maintained color more effectively on fresh-cut cantaloupe and apple slices. Exopolysaccharides produced by Xanthomonas campestris under adverse conditions are considered generally recognized as safe (GRAS) substances according to FDA regulations [26]. The inoculation of fresh-cut cantaloupe with *L. plantarum B2* aided the preservation of the physicochemical and nutritional qualities of the melons, except for some alterations in the sensorial quality attributes of the fruit [27]. Sharma & Rao. [28] reported that the incorporation of cinnamic acid as an antioxidant agent into xanthan gum-based edible coating caused significant retardation of the oxidative browning and a decline in the ascorbic acid level, degradation of total phenolics content, and reduction in the antioxidant capacity of fresh-cut pears as compared to those coated only with xanthan gum and the uncoated. Similarly, reduced weight loss and oxidative browning, increased fruit firmness, and lower growth of psychrotrophic and pathogenic microorganisms were reported when Xanthan gum was used as a carrier of preservatives and calcium chloride in an edible coating applied on fresh-cut apples [29]. Similarly, fresh-cut kiwi fruit retained higher ascorbic acid, total phenolic components, and antioxidant capacity levels after coating with xanthan polysaccharide [30]. Therefore, the novel approach to preserving the quality of fresh-cut melons and enhancing their functional benefits could be through loading edible natural polysaccharides (xanthan gum) with probiotic bacteria. Thus, the objective of this study was to assess the effects of xanthan gum and lactic acid bacteria coatings (probiotic coatings) on postharvest quality, microbial quality, retention of bioactive compounds, and acceptability after 5 days at 5 °C of storage of fresh-cut Honeydew (*Cucumis melo* L.) and cantaloupe (*C. melo* var. *cantalupensis)* melons. 

## 2. Materials and Methods

### 2.1. Chemicals

All chemicals used in this study were purchased from Sigma Aldrich (Johannesburg, South Africa) and were of analytical grade.

### 2.2. Melon Fresh Cut

At commercial maturity, cantaloupe and honeydew melons were harvested from the Hygrotech Experimental Farm. ‘Majestic’ cantaloupe melons were picked 10 weeks after transplanting when their skin turned golden yellow and formed a well-rounded, corky net with a musky smell. The ‘Honeyval’ honeydew melons were harvested once their skins had turned white. A total of 15 fruits were harvested per cultivar that had the same size and no defects from a commercial farm (Pretoria, South Africa) during the 2022 to 2023 growing seasons [31]. The fruits were transported to the laboratory for analysis of the fresh-cut melons. Upon receiving melons, the epidermis (skin) was brushed with a brush to remove soil and dirt. We selected melons without flesh damage or discoloration; all cubes of intact quality were sanitized, and all cubes of damaged quality were rejected. A solution of Clorox [5.25% sodium hypochlorite (NaOCl), Clorox Africa (Pty) Ltd., Johannesburg, South Africa] was used to surface sterilize the melons, and sterile water was used to wash them. The melon slices were cut parallel to the longitudinal axis, and the blossom stem ends, seeds, and placentas were discarded. Thereafter, the melons were peeled and cut into two halves, and the seeds were manually removed using stainless steel knives under sterile conditions. Stainless steel knives were used to cut the melon into cube pieces (1.5 cm × 1.5 cm). 

### 2.3. Probiotic Cultures

The *Lactiplantibacillus plantarum* 75 (LAB 75) was obtained from the University of Reunion Island, France, while the *Bifidobacterium longum* strain was marketed commercially from Biokar Diagnostics (Solabia group, Pantin, France). These cultures were activated overnight in de Mann, Rogosa Sharpe (MRS) broth and later in MRS broth supplemented with L-cysteine HCL and D-glucose. They were sub-cultured in MRS media till a pure colony was obtained. Overnight-grown LAB culture cells were harvested by centrifugation and washed in sterile saline water. The cells were suspended in water, and the concentration was adjusted to 0.5 optical density (10^8^ CFU/mL) using a UV-visible spectrophotometer. 

### 2.4. Coating Preparation

To prepare the edible coatings, xanthan gum solution and 2.5 g of powdered xanthan gum were mixed with 1 L of autoclaved distilled water at 50 °C. Citric acid [(2% (*w*/*w*)] antibrowning agent and 1% (*w*/*w*) glycerol plasticizer were then incorporated into the xanthan gum solution as described by Lara et al. [32]. 

### 2.5. Coating of Melon Fresh Cuts

The fresh-cut melon cubes (‘Majestic’ cantaloupe and ‘Honeyval’ honeydew melons) were coated with xanthan gum, xanthan—*Lactiplantibacillus plantarum* 75, and xanthan- *Bifidobacterium longum* suspension by dipping for 3 min. Thereafter, the coated fresh-cut melons were kept in a laminar flow hood, drying for 30 min at 20 °C. After coating, the coated melons were aseptically packed into LOCK&LOCK fresh-keeping boxes (polypropylene, 383 × 320 × 93 mm) and stored at 5 °C for 5 days to simulate the commercial storage time and temperature. Ten punnet replicates, each containing cantaloupe and honeydew fresh-cut melons (with 12 cubes each weighing 250 g) in separate containers, were included in all treatments. A control group consisted of fresh melon cuts without probiotic coating, fresh melon cuts coated with xanthan, and uncoated melon fresh cuts dipped in deionized water. 

**Figure 1:**
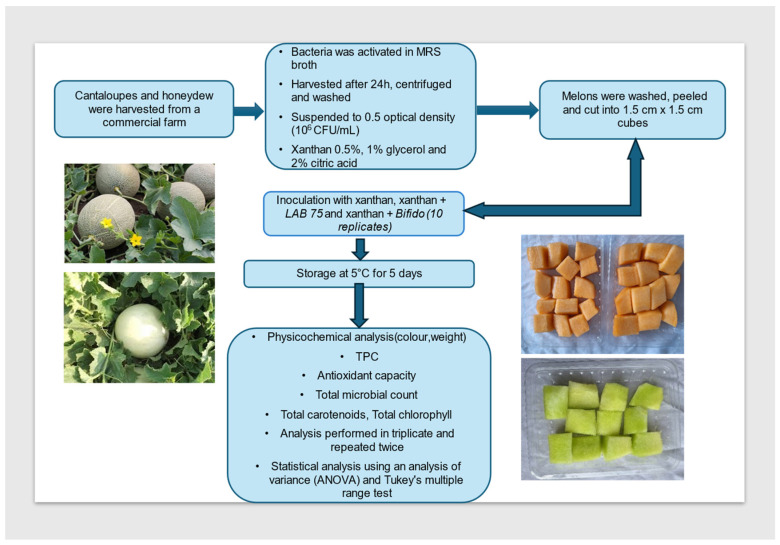
Process flowchart of fresh-cut cantaloupe and honeydew (*Cucumis melo* L.) melons coated with probiotic bacteria.

The following treatments were included in this study: (1) cantaloupe (‘Majestic’) or ‘Honeyval’ honeydew melons coated with xanthan gum, (2) cantaloupe (‘Majestic’) or ‘Honeyval’ honeydew melons coated with xanthan—*Lactiplantibacillus plantarum* 75, (3) cantaloupe (‘Majestic’) or ‘Honeyval’ honeydew melons coated with xanthan- *Bifidobacterium longum*, (4) uncoated cantaloupe (‘Majestic’) or ‘Honeyval’ honeydew melons, (5) freshly cut cantaloupe (‘Majestic’). The microbial quality evaluation was conducted on day 5 with three replicates per treatment. The remaining seven replicates were used to analyze physicochemical parameters, organoleptic properties, and changes in antioxidant components and their activities. Samples to analyze the antioxidant components and properties were snap-frozen in liquid nitrogen and stored at −80 °C for further analysis. The methods and processes followed in this study are illustrated in Figure 1.

### 2.6. Coating Characterization

The coating was prepared according to the method illustrated in Section 2.4. However, it was further dried in the oven at 25 °C for 7 days. 

FTIR spectra were acquired using a Perkin Elmer Spectrum 100 FTIR spectrometer (Perkin Elmer Waltham, MA, USA) with the Miracle ATR attachment and a Zn/Se crystal at a spectral resolution of 4 cm^−1^. The FTIR measurements were performed with a scan range of 4.000–600 cm^−1^ [33]. 

TGA (TGA Q500, TA instruments, New Castle, DE, USA) was used to determine the thermal stability of the various samples with a temperature ramp from room temperature to 900 °C with a ramp of 10 °C/min in an air atmosphere with a flow rate of 60 mL/min [34]. 

Rheological measurements were conducted using a controlled-strain rheometer (ARES-G2, TA Instruments, New Castle, DE, USA) with a parallel plate geometry (40 mm diameter, 1 mm gap). Small-amplitude oscillatory shear (SAOS) measurements were performed within the linear viscoelasticity regime at frequencies ranging from 10^−2^ to 10^2^ ad/s [35]. 

The mechanical properties of tensile strength and elongation were measured using a texturometer (TA. XT Plus Texture Analyser, Stable Micro Systems, New Castle, DE, USA) according to de Morais Lima et al. [36]. 

The zeta potential and the polydispersity index (PDI) were analyzed using dynamic light scattering (DLS) analysis at 25 °C (Zetasizer Nano ZS-90, Malvern Instruments, UK) using a folded capillary cell (DTS 1060, Malvern Instruments) following a method illustrated by Osondu et al. [33]. 

### 2.7. Survival of LABs and Microbial Count

The evaluation of the total viable count and surviving LAB count of the coated and uncoated melons was determined using the pour plating techniques [37]. The serially diluted mixture obtained from crushed melon samples was plated on appropriate media. The MRS plates were incubated at 30 °C for 48 h. The surviving LAB, aerobic bacterial counts were enumerated using a digital colony counter, and results were expressed as logarithmic colony-forming units per gram (Log CFU/g) of the sample.

### 2.8. Postharvest Quality

#### 2.8.1. Color Analysis

The color of fresh-cut melons was evaluated using a spectrophotometer (CM-5, Konica Minolta, Tokyo, Japan) before and after 5 days of storage at 5 °C. The color was measured according to the CIE L*a*b* values recorded from the spectrophotometer. The L* represents the lightness value (0 = black, 100 = white), a* the red/green value (+value = redness, −value = greenness), and b* the yellow/blue value (+value = yellowness, −value = blueish) [38].

#### 2.8.2. Sensory Analysis

A quantitative descriptive analysis technique, as described by Menezes Ayres et al. [39], was used for the sensory evaluation of the fresh-cut melons, with some modifications. Ten trained panelists were selected from the pool of assessors trained to identify the desired characteristics of the melons. The panelists were composed of healthy males and females. Two training sections were adopted, and the samples were rated using a structured scale ranging from 0 to 9 (Absent = 0, 1–3 = weak, 4–6 = moderate, 7–9 = strong). Panelists evaluated samples based on the agreed attributes of the melons, and the ratings of samples were converted into intensity scores. To evaluate the color perception of cantaloupes, the participants were asked to determine whether the color of the tangerine peel was bright or dark orange. Similarly, for honeydew melons, the perception of bright, light, or dark green color with reference to green apple peel was used. The aroma of cantaloupe was found to be similar to that of watermelons, while the aroma of honeydew was described as fruity and musky. The sweetness of the melons was compared to the sweetness of a 70% sucrose solution, and the firmness was evaluated based on the degree of compression when biting into a fresh, ripe apricot (strong) or an overripe mango (weak). The sourness was assessed using unripe apple slices, and the crispness was evaluated based on the hard texture perceived when biting into the melon compared to freshly cut pieces. The juiciness was referenced to the water released upon biting into the melon, as is typical of fresh-cut watermelon.

### 2.9. Changes in Bioactive Compounds and Antioxidant Activities of Coated Fresh-Cut Melons

#### 2.9.1. Ascorbic Acid

The ascorbic acid (AA) content of the fruit was then determined using the 2,6 dichlorophenolindophenol (DCIP) titration method as described by Mphaphuli et al. [40] using a fresh fruit sample of 5 g macerated in 50 mL of 3% cold metaphosphoric acid (HPO_3_). The calibration curve was calculated using standard L-ascorbic acid (Sigma-Aldrich, Johannesburg, South Africa), and the results were expressed in mg of ascorbic acid per 100 g fresh weight (FW).

#### 2.9.2. Total Phenolic Content

The total phenolic contents of the coated and uncoated fresh-cut melons were determined using the Folin–Ciocalteu reagent [41]. The total phenolic content was calculated using a standard curve of gallic acid and expressed as mg of gallic acid equivalents per 100 g fresh-cut fruit.

#### 2.9.3. Total Carotenoid Content

The carotenoid extract of cantaloupe melon was analyzed using spectrophotometry at wavelengths ranging from 250 to 700 nm. Using the equation of Biehler et al. [42] at the wavelength of maximum absorption, the mean carotenoid concentration was calculated: C (mol/L) = A450 × FD/2592 (d = 1 cm), A450 being the average absorbance value at this wavelength, FD is the dilution factor corrected for the absorbance readings of the hexane dissolving dry extract, and 2592 is the coefficient of absorption (ε) of β-carotene. The result was calculated in μg/g of melon pulp.

#### 2.9.4. The Total Chlorophyll Content

The chlorophyll contents were analyzed according to the method described by Managa et al. [43]. 0.2 g of ground samples was mixed with 2 mL acetone and hexane 4:6 (*v*/*v*). The slurry was centrifuged for 10 min at 4 °C (9558× *g*), the supernatant was plated on a 96-well plate, and the absorbance was read at 470, 646, and 662 nm (Biochrom Anthos Zenyth 200 Microplate Reader; SMM Instruments, Biochrom Ltd., Johannesburg, South Africa). The concentration of each pigment was calculated according to the formula below and was reported in mg 100 g^−1^ on the dry weight of the sample.

Chlorophyll a = 15.65 (A_662_) − 7.34(A_646_). (1)

Chlorophyll b = 27.05(A_646_) − 11.21(A_662_). (2)

Total chlorophyll = Chlorophyll a + chlorophyll b(3)

### 2.10. Antioxidant Activities

#### 2.10.1. Ferric Reducing Antioxidant Power (FRAP)

The ferric-reducing antioxidant activity was quantified according to the procedure described by Ndou et al. [44]. The absorbance was measured at 593 nm using the spectrophotometer. Trolox solution ranging from 10 to 1000 mg/mL was prepared and used to develop the calibration curve. 

#### 2.10.2. 2,2-Diphenyl-1-picrylhydrazyl (DPPH)

The radical scavenging activity was assessed using the DPPH scavenging activity assay according to the method described by Seke et al. [41]. The absorbance was measured at 517 nm after incubating for 20 min at room temperature in a microplate reader. The concentration of antioxidants was calculated as _IC50_ from the graph showing the antioxidant inhibition percentage versus the concentration.

#### 2.10.3. 2,2-Azinobis-(3-ethylbenzothiazoline-6-sulfonate) (ABTS) Assay

A method described by Managa et al. [43] was used to determine ABTS^+^ radical scavenging assay. The ABTS^+^ scavenging activity was then calculated at 734 nm. The sample concentration providing 50% inhibition (IC_50_) was calculated from the inhibition percentage versus the concentration graph.

### 2.11. Statistical Analysis

A completely randomized design was employed with a set of ten replicate punnets in the experiment. The experiments were repeated twice, and the data were analyzed using one-way ANOVA in the statistical program Statistica data analysis software system (10) (Statsoft, Inc., Tulsa, OK, USA). The Fisher LSD test was performed to discover significant differences at *p*-values < 0.05. The correlation between the phenolics and antioxidant properties was established using Pearson regression correlation coefficients. 

## 3. Results and Discussion 

### 3.1. Characterization of Xanthan Gum-Based Edible Coating 

#### 3.1.1. Zeta Potential, Particle Size and Polydispersity Index (PDI) for Pure Xanthan Gum, Xanthan Gum + *L. plantarum 75*, and Xanthan Gum + *Bifidobacterium longum* Coating

The values of the zeta potential, z-average, and PDI of pure xanthan gum, xanthan gum + *L. plantarum 75,* and xanthan gum + *B. longum* coating are demonstrated in Table 1. As can be seen, the z-average values of samples increased when *L. plantarum 75* (498.1 nm) and *B. longum* (641.5 nm) were added to the xanthan gum (420.4 nm) system. The zeta potential value increased slightly when *L. plantarum 75* was added to the pure xanthan system. Contrarily, the zeta potential values decreased significantly from −50.5 to −39.7 mV with the addition of *B. longum*. The changes in zeta potential may be caused by the alteration of electrostatic repulsion and interfacial interaction between xanthan gum, *L. plantarum 75,* and *B. longum*. This decrease in the zeta potential indicates that the xanthan gum polysaccharide matrix, through either the interaction of different functional groups with bacterial surface or aggregation within the dispersion, resulted in the perturbation of the surface charge density of the *B. longum* [45]. It is known that dispersion or suspension with a low zeta potential value promotes the aggregation of particles due to van der Waals attractions [46], which was also observed in this case as confirmed by the higher z-average value. However, this did not affect the stability of the dispersion as the absolute value above 25 mV and the zeta potential is generally considered an indication that the particle suspension will be electrostatically stabilized [47]. The PDI showed to be stable throughout all the coating systems, showing a PDI value of 1. A similar PDI of 1.014 has been reported by Faria et al. [48]. In the absence of other limiting variables like high viscosity and pH, colloids containing particles with low zeta potential (positive or negative) will have a strong inclination to coalesce and agglomerate. Positive charges and zeta potential will grow in an acidic environment, and the converse is true in an alkaline one because of pH.

#### 3.1.2. Rheological Properties of Pure Xanthan Gum, Xanthan Gum + *L. plantarum 75*, and Xanthan Gum + *Bifidobacterium longum* Coating

**Figure 2:**
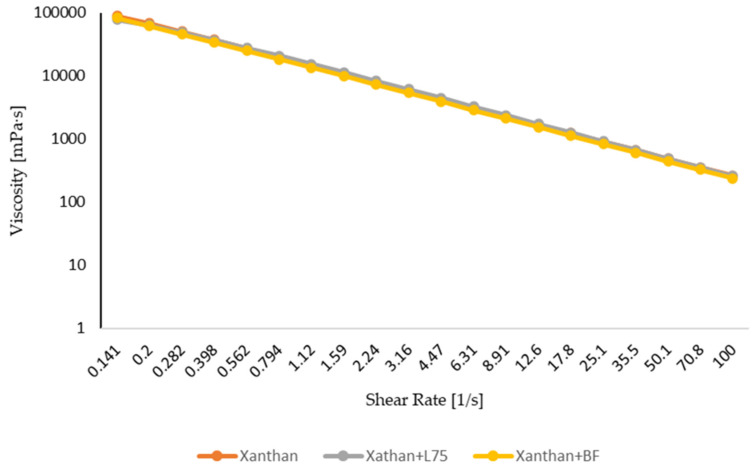
Rheological properties (viscosity) of xanthan pure xanthan gum, xanthan gum + *L. plantarum 75* (xanthan + L75), and xanthan gum + *Bifidobacterium longum* (xanthan + BF) coating.

The apparent viscosity of the three solutions, pure xanthan gum, xanthan gum with *L. plantarum 75*, and xanthan gum with *B. longum,* is shown in Figure 2. All of the samples showed the typical pseudoplastic behavior, in which the viscosity curves fall as the shear rate increases. Similar results were observed by Miranda et al. [49] in research on the influence of *Xanthomonas* spp. on the properties of xanthan gum. Research on the effects of mixing xanthan gum with cheese whey, fructooligosaccharide, and *Lacticaseibacillus casei* CSL3 also yielded similar results [50]. The viscosity measurements make it clear that the addition of lactic acid bacteria did not affect the pure xanthan gum’s viscosity. Dario et al. [51] claim that at high shear rates, in addition to chain position, the bonds connecting these can break, rupturing the bonds that link the material’s structural components and producing low viscosities. At high concentrations, the apparent viscosity rises in proportion to the entanglements of macromolecular chains. The orientation of macromolecules along the streamline of the flow is connected to the phenomena of shear thinning behavior [52]. The significant fluid flow resistance causes the high viscosity, whereas the low shear rate is caused by the stretched polysaccharide molecules intertwining to form aggregates [53]. A decrease in the flow resistance of fluids, the destruction of aggregates, and the dispersing of molecules oriented along the flow direction are the results of increasing shear rate, which also lowers apparent viscosity [54]. In the context of creating a protective layer over the core material, the samples’ increased viscosity may be advantageous. This property becomes especially useful when fruit and vegetable samples are coated with the coating and pass through the digestive system because it can lower the pace at which bile salts and acid medium enter microcapsules. The coating can improve probiotic bacterial and nutritional component preservation and survival by slowing down the transit of these harsh chemicals [55,56].

#### 3.1.3. Mechanical Properties (Tensile Strength) of Pure Xanthan Gum, Xanthan Gum + *L. plantarum 75*, and Xanthan Gum + *Bifidobacterium* Coating

The results obtained for tensile strength determination for all the coating formulations are shown in Table 2. Tensile strength (TS) significantly decreased (*p* < 0.05) from pure xanthan gum from 1 ± 0.4 MPa to 0.74 ± 0.2 and 0.83 ± 0.4 MPa, respectively, for xanthan gum + *L. plantarum 75* and xanthan gum + *B. longum* coating. The reduction suggested a lowering of intermolecular interactions and was compatible with the addition of the lactic acid bacteria. There is a great possibility that the lactic acid bacteria in the current study might have changed the molecular structure of the films’ matrix, destroyed its surface, and enlarged the intermolecular space of the film’s surface, decreasing the tensile strength of the edible coatings [57]. Ebrahimi et al. [58] also reported a decrease in tensile strength with the addition of Lactic acid bacteria (*Lactobacillus acidophilus*, *L. casei*, *L. rhamnosus,* and *Bifidobacterium bifidum*) in carboxymethyl cellulose-based edible film. It has been reported that probiotic cells can interrupt the cohesiveness of the polymer chains, thereby weakening the edible coating [59]. The greatest intensity FTIR finding was produced by the presence of the OH group (water) in xanthan gum, which also probably decreased the film’s stiffness and resistance to elastic force, lowering the composites’ tensile strength [59]. This finding is consistent with research by Jia et al. [60] that discovered that adding konjac glucomannan-chitosan edible coating to soy protein isolate reduced the tensile strength by weakening the intermolecular forces between the film’s constituent molecules. 

#### 3.1.4. Thermal Stability of Xanthan Gum, Xanthan Gum + *L. plantarum 75*, and Xanthan Gum + *Bifidobacterium longum* Coating

**Figure 3:**
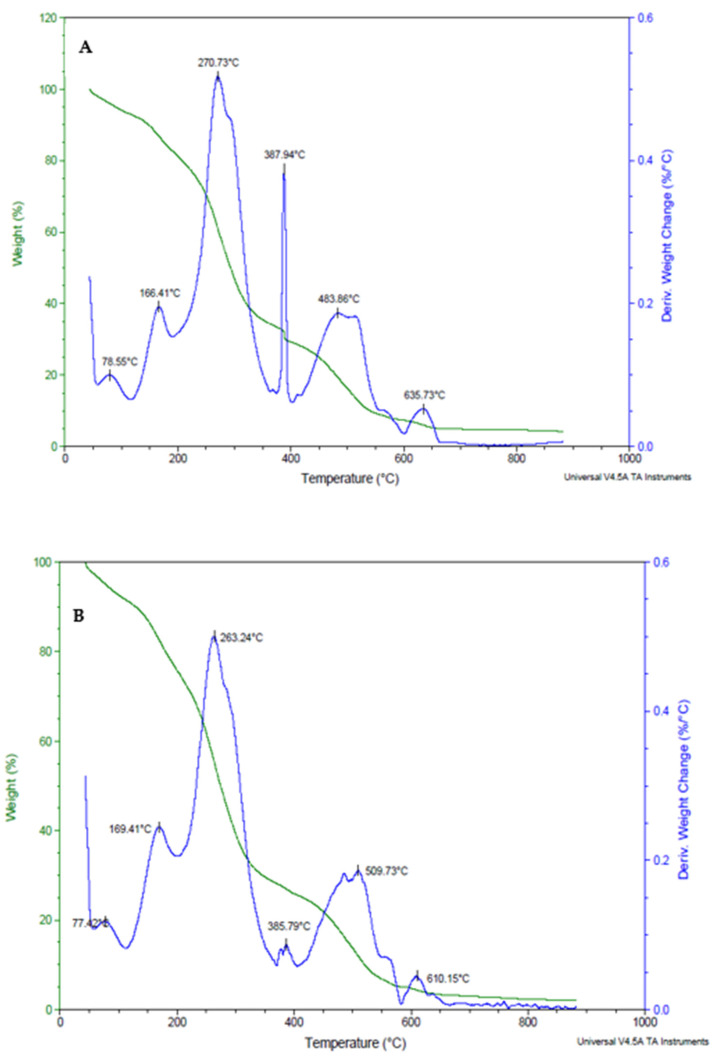

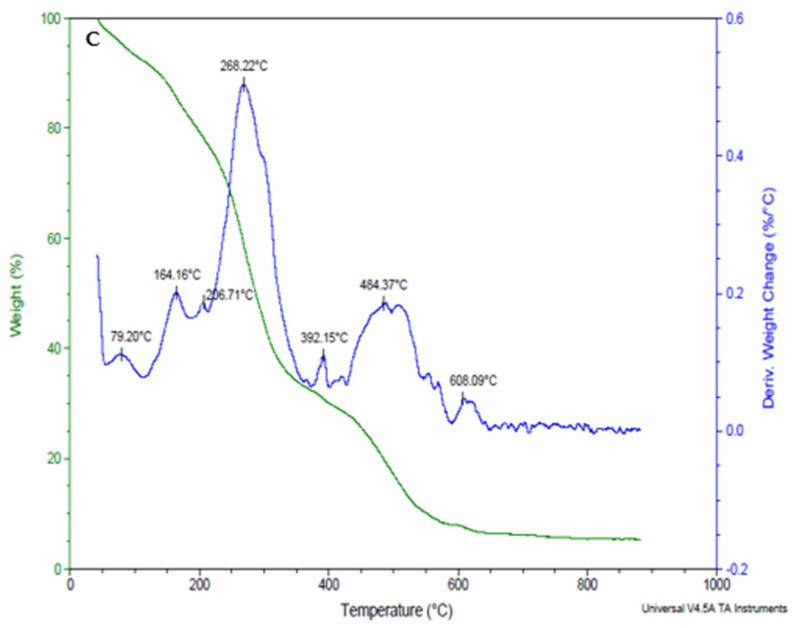
The thermal stability of pure xanthan gum, xanthan gum + *L. plantarum 75,* and xanthan gum + *Bifidobacterium longum* coating. (**A**): pure xanthan gum, (**B**): xanthan gum + *L. plantarum 75,* and (**C**): xanthan gum + *Bifidobacterium longum* coating.

**Figure 4:**
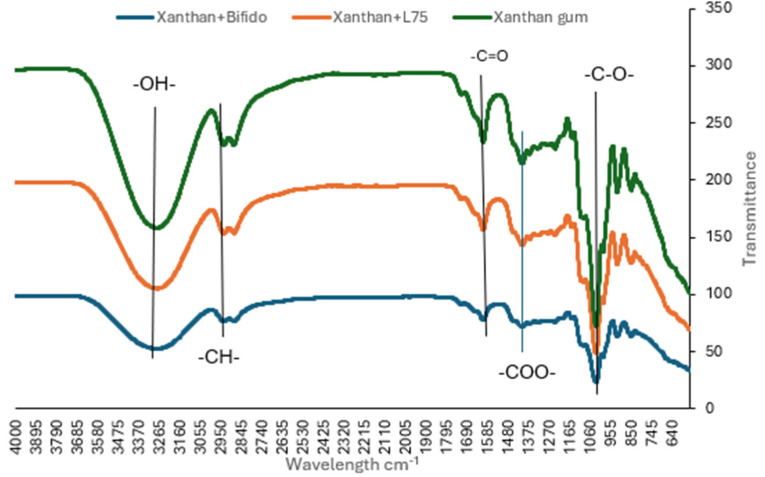
FTIR of pure xanthan gum, xanthan gum + *L. plantarum 75,* and xanthan gum + *Bifidobacterium longum* coating.

Figure 3A–C shows the weight loss of pure xanthan gum and xanthan gum edible coating incorporating *B. longum* and *L. plantarum 75* microorganisms. The weight loss of xanthan gum, xanthan gum + *L. plantarum 75*, and xanthan gum + *B. longum* (Figure 4) was seen to be between 10 and 15% up until 100 °C, as indicated by the thermograms. This phenomenon may be explained by the material’s adsorption of water. Before the thermal breakdown, which happened above 200 °C in a single, sudden step and resulted in a weight loss of between 40% and 50%, a plateau was seen in both thermograms. After 380 °C, the thermal breakdown continued steadily at a reduced pace until it reached around 950 °C. A common phenomenon for every film/edible coating is that the initial weight loss is the loss of adsorbed water followed by the dehydroxylation of the biopolymers and glycerol, and then organic matter breaking down. After 150 °C, there was a greater degree of dehydroxylation of the hydrogen-bonded organic components (Figure 2). Lawal et al. [61] state that the temperature range between 120 and 300 °C is where glycerol breaks down. Most likely, organic component dehydroxylation began earlier than 120 °C. It is thought that the dehydroxylation process can be induced at reduced temperatures by roughly matching the chains of organic components in the edible coating with the removal of adsorbed water. De Morais Lima et al. [30] observed that there was no plateau of thermal stability before the thermal breakdown of the organic material, indicating that weight loss likely began before completing the dehydroxylation stage. Compared to pure biopolymers, the distinct thermogram profiles of the edible coating suggest a considerable interaction between the organic components. For instance, the polar side chains of xanthan gum and lower chains of glycerol contribute to the breakdown of organic matter at lower temperatures, possibly occurring in stages. According to Soares et al. [62], the side chains broke first in the breakdown of xanthan gum and then the main chain. During the analysis, it is possible to see a continuous and multi-step weight reduction in this work, illustrating how the edible coating components interact.

#### 3.1.5. FTIR of Pure Xanthan Gum, Xanthan Gum + *L. plantarum 75* and Xanthan Gum + *Bifidobacterium longum* coating

The infra-red vibrational bands that are characteristic of the xanthan gum with or without *L. plantarum 75* or *B. longum* (Figure 4) provide valuable information about the polymer structure, so FTIR spectroscopy was used to examine the existence of possible reactions between xanthan gum and the two different bacteria. The analyzed samples showed the same band and vibration patterns. The bands presented between 3500 and 2951 cm^−1^ were relative to O single bond H stretching vibrations with intermolecular H-bonding and C single bond H stretching, respectively. The most important was relative to the -COCO single bond (C single bond O stretching coupled to adjacent C single bond C stretching) vibrations from acetal rings at 1020 cm^−1^ from xanthan gum. Similar bands have been reported for other commercial gums, such as guar and xanthan. It was concluded that the bands around 3400 cm^−1^, 2939 cm^−1^, and 990–1200 cm^−1^ are common to all polysaccharides, and they represent O–H bonds, C–H bonds of CH_2_ groups and saccharides, respectively [48]. Similar bonds have been reported in a study on the FTIR and impedance study of xanthan gum as a host matrix for the ionically conducting membranes [63]. The shift in the band patterns observed can be attributed to the hydrogen bonds and interactions between molecules within the biopolymers [64]. However, the FTIR spectra for xanthan gum and *B. longum* showed a more negative result as the xanthan gum bonds seemed to have reduced intensity. This can be attributed to interactions between components or even to dilution effects (by adding other components) of the film system. A similar trend in the decrease in intensity has also been reported by Oliveira-Alcântara et al. [65].

### 3.2. Survival of Probiotic Bacteria in Edible Coating 

**Figure 5:**
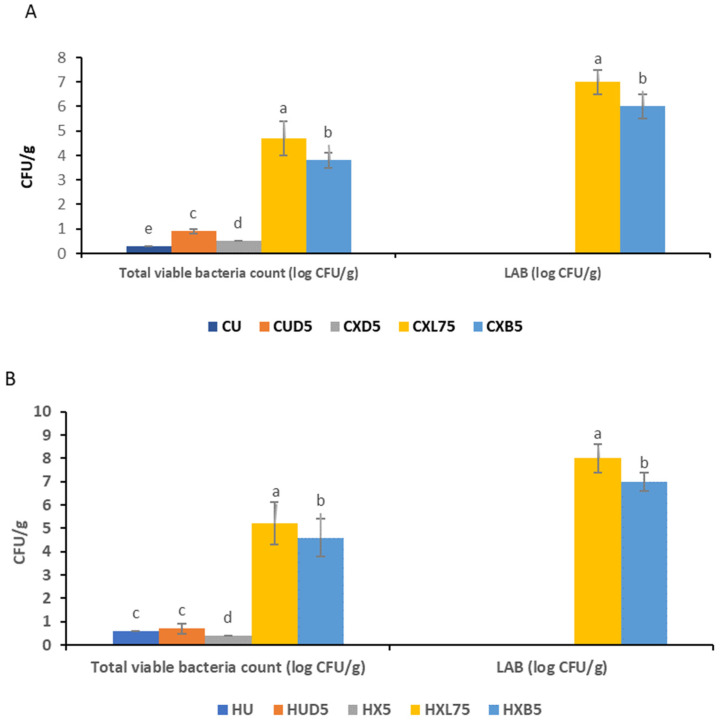
Effect of coating on the total viable bacterial, lactic acid bacteria, of coated and uncoated melons, (**A**): Cantaloupe melons, (**B**): Honeydew melons. Bars represent means, and error bars represent the standard deviations from the mean. Alphabets on the same-colored bar with different letters are significant (*p* < 0.05). CU = uncoated cantaloupe, CUD5 = uncoated cantaloupe fresh cuts, CXD5 = xanthan coated cantaloupe fresh cuts, CXL75 = Xanthan-*L. plantarum* LAB 75 coated cantaloupe fresh cuts and CXB5 = Xanthan-*Bifidobacterium longum* coated cantaloupe fresh cuts, HU = uncoated honeydew melon fresh cuts, HUD5 = uncoated honeydew melon fresh cuts, HX5 = xanthan coated honeydew melon fresh cuts, HXL75 = Xanthan-*L. plantarum* LAB 75 coated honeydew melons fresh cuts and HXB5 = Xanthan-*Bifidobacterium longum* coated honeydew melon fresh cuts.

Figure 5 demonstrates the viability of *Lactobacillus* strains in xanthan coating while storing cantaloupe and honeydew melons at 5 °C for 5 days. In cantaloupe, fresh cuts coated with CXL75 coating, *L. plantarum 75* (LAB *75*) counts were significantly higher at 7 log CFU g^−1^, while *B. longum* (CXBD5) survived at 6 log CFU g^−1^. Similarly, in honeydew melon fresh cuts, *L. plantarum 75* (LAB *75*) (HXL75) survived at 8 log CFU g^−1^, while *B. longum* (HXBD5) survived at 6 log CFU g^−1^. It has been found that the effectiveness of probiotics in food depends on the concentration and survival of the added cells [66]. To ensure that food products contain enough live probiotics, the food industry has set a minimum standard of 6 log CFU g^−1^. Therefore, honeydew melons and cantaloupes coated with xanthan and *L. plantarum 75* or *B. longum* have been shown to have probiotic potential. Recent studies have shown that certain food coatings can contain probiotics in levels far exceeding the minimum threshold of 6 log CFU g^−1^. Although various factors influence the stability and growth of these probiotic bacteria, the use of lock and lock containers prevented the negative effects that could have affected the viability of these bacteria. The usage of low temperatures for storage prevented the loss of the LABs, as high temperatures decrease microorganisms’ viability. Low temperatures have been reported to aid in better survival of certain probiotics [67], and exposure to oxygen, on the other hand, has a detrimental effect on bacterial survival as well [68]. Uncontrolled environments are associated with a decline in pH, and extremely low pH is associated with a reduction in growth yield because it can lead to undissociated acids [69]. For instance, strawberries coated with carboxymethyl cellulose containing *L. plantarum* retained viable probiotics (higher than 6 log CFU g^−1^) even after 15 days of storage at 4 °C [23]. Similarly, fresh-cut yacon (*Smallanthus sonchifolius*) coated with the mucilage of linseed (*Linum usitatissimum*) film-forming solution that contained *L. casei* LC-01 showed a survival rate of around 8 log CFU g^−1^ after 15 days of storage at 5 °C [70]. Fresh-cut papayas and apples coated with alginate and gellan-based edible coatings carrying *B. lactis* Bb-12 and stored for 10 days at 2 °C showed 6.24 to 7.31 log CFU g^−1^ [22]. Finally, fresh-cut kiwis coated with *L. plantarum* strains (*L. plantarum* LP3, *L. plantarum* AF1, and *L. plantarum* LU5) incorporated into a Konjac-based edible coating and stored at 4 °C for 5 days showed a retention rate of 7.1, 6.4 and 6.5 log CFU g^−1^ [71]. Thang et al. [72] highlighted that incorporating Xanthan gum and alginate provided a protective effect on the viability of *L. acidophilus* in bread, especially under simulated gastric and intestinal conditions, compared to using alginate alone. Although the lock and lock containers protected the samples from oxygen exposure, oxygen could not completely be eliminated from the container, as well as the changes that occur when the samples are stored at 5 °C and above. In the future, vacuum sealing can be suggested for this kind of study, and lower temperatures, such as −24 and 2 °C, can be utilized [69]. Screening probiotic bacteria for oxygen tolerance before their incorporation could ensure high cell counts in food products during storage [73]. When used alone, xanthan gum is prone to microbial contamination, exhibits unstable viscosity, and has an uncontrollable hydration rate [74]. Additionally, it results in gels with inadequate shear resistance, mechanical strength, and thermal properties. To improve the coating properties of xanthan gum in probiotic encapsulation, it is often combined and utilized in conjunction with other biomaterials such as alginate, chitosan, gellan, and β-cyclodextrin [74]. 

### 3.3. Probiotic Coatings and Color Properties

Consumers’ decision-making when buying products can be affected by the product’s visual appearance [75]. To test the probiotic coating’s effect on color, the statistical hypothesis was tested on the color properties L (Lightness), a (green/red and b (yellow/blue coordinate) [76]. The different treatments applied to the fresh cuts of melon determined all three color coordinates. The lightness (L*) values in cantaloupe fresh-cut melons declined throughout the storage of the coated and uncoated melons (Table 3). However, Xanthan-LAB 75 (CXL75) retained lightness better in cantaloupe fresh-cut melons. Fresh-cut honeydew melons were protected from loss of light by HXL75 and HXB5 coatings. Uncoated cantaloupe (CUD5) and honeydew (HUD5) melon fresh cuts showed darker flesh color due to loss of lightness. Reduced luminosity observed in uncoated melons may be associated with polyphenol oxidase activation, which causes enzymatic browning due to physiological damage. In contrast, the coated samples showed reduced browning due to reduced oxygen penetration, reducing oxidation rates [77]. Moreover, the probiotic-loaded edible coating or coatings prevent the enzymatic oxidation of phenolic compounds, thereby protecting them [71]. Observations showed that the a* value in uncoated melons was 9.72, and on the fifth day, the a* value increased to 14.52, probably due to the initiation of browning. Fresh-cut cantaloupe melon coated with CXL75 showed a lower color coordinate, showing less browning. However, HUD5 showed a slight increase in value (−0.46), followed by HXB5 (−1.26) and HXL75 (1.38). HXB5 showed a slight deviation in color coordinates compared to HU (freshly cut honeydew melons on d 0). CXL75 had higher b* values compared to uncoated samples; hence, *L*. *plantarum 75* enhanced the preservation of the yellowness parameters in fresh-cut cantaloupe melons after 5 days of storage. Cantaloupe fresh cuts at day 5 cold storage (CUD5) became darker and had a less intense yellow color. Honeydew melons coated with *B. longum* (HXB5)- retained their flesh color (b*) in comparison to the other treatments. Thus, probiotic coatings likely delay color deterioration during storage by delaying oxidative processes and pigment metabolism (chlorophyll degradation and carotene synthesis) [8]. The alginate coating containing *L. plantarum* c19 preserved the color of the melon and apple slices during storage [25]. Furthermore, the inclusion of probiotic cells in coatings or edible coating prevents moisture loss and color changes by forming hydrogen bonds between bacteria and coating components [78]. As seen with probiotic-coated Kiwi fruits, incorporating probiotics in coatings could have inhibited fungal growth, leading to less color change [75].

### 3.4. Probiotic Coatings and Sensory Properties

**Figure 6:**
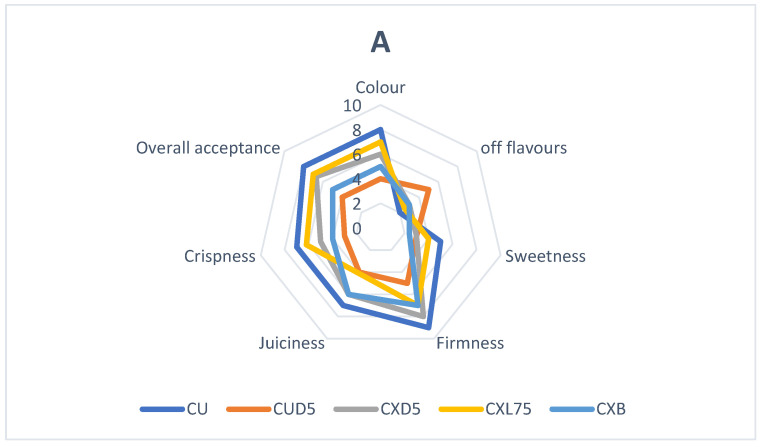

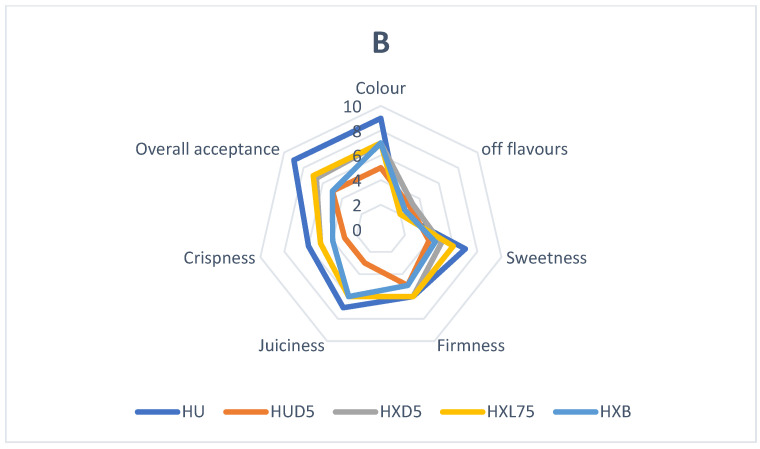
Effect of coating on the sensory properties of cantaloupe (**A**) and honeydew (**B**) melons after 5 days of storage. CU = uncoated cantaloupe at day 1, CUD5 = uncoated cantaloupe stored for 5 days, CXD5 = Xanthan coated cantaloupe stored for 5 days, CXL75 = Xanthan-*L. plantarum* LAB 75 coated cantaloupe at day 5 and CXB5 = Xanthan-Bifidobacterium longum coated cantaloupe stored for 5 days), HU = uncoated honeydew, HUD5 = uncoated honeydew stored for 5 days, HXD5 = Xanthan coated honeydew stored for 5 days, HXL75 = Xanthan-*L. plantarum* LAB 75 coated honeydew melons stored for 5 days, and HXB5 = Xanthan-*Bifidobacterium longum* coated honeydew melon fresh cuts.

**Figure 7:**
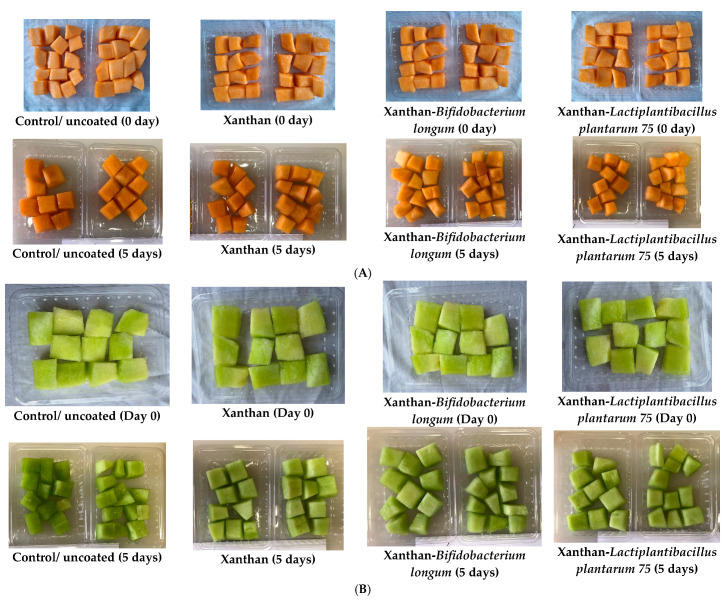
(**A**) Appearance images of fresh-cut cantaloupe melon covered with different films during five days of storage. (**B**) Appearance images of fresh-cut honeydew melon covered with different films during five days of storage.

Figure 6A, B illustrates the organoleptic properties of fresh-cut cantaloupes and honeydew melons. CU samples are firmer, crispier, and juicier than the other samples. The panelists said that CXL75-coated fresh cuts displayed superior sweetness and color attributes. Gänzle et al. [79] stated that sucrose metabolism by probiotic bacteria may be responsible for the increase in sweetness observed in HXL75 and CXL75-coated fruits. Russo et al. [15] previously showed that cantaloupe melon fresh cuts coated with *L. plantarum* B2 displayed glucose and fructose evolution during storage time. CXL75 showed the highest color retention, sweetness, firmness, crispiness, and overall acceptance (Figure 5A). Regarding the honeydew (HU) fresh cuts, the panelists concluded that samples coated with HXL75 were firmer, crispier, and displayed higher color retention and overall acceptance than the treatments BD5, XD5, and uncoated fruits (HU). The color parameters (L* a* and b*) may have contributed to the higher perception of the color. Further, no off-flavors were detected in the probiotic-coated cantaloupes or honeydew melons, suggesting that a short shelf life of up to 5 days should not adversely affect sensory quality. However, Jacxsens et al. [80] stated that the sensory properties of fresh-cut produce could be negatively impacted by the colonization of lactic acid bacteria by producing carbon dioxide, ethanol, organic acids, and volatile esters. The results suggest that adding probiotics to edible coatings maintained the sensory properties of the fresh-cut melons during storage. However, the selection of probiotic strains differed based on different melon varieties. Figure 7A, B illustrates the overall appearance of the fresh-cut honeydew and cantaloupe melons at 0 days and at after 5 days of cold storage. 

### 3.5. Probiotic Coatings and Total Carotenoids and Chlorophylls 

**Figure 8:**
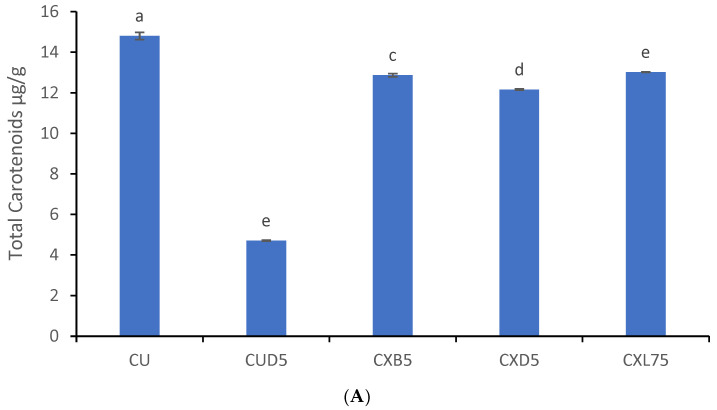

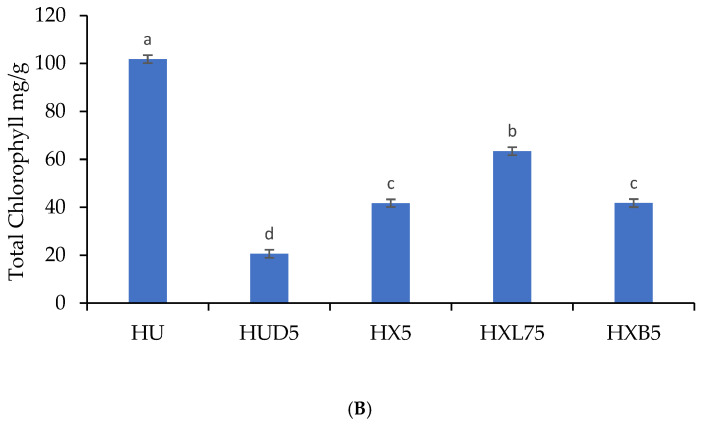
(**A**). Effect of coating on total carotenoids in cantaloupe melons. Data is presented as means, and error bars are standard deviations from the mean. Bars for each melon cultivar, with different letters, are significantly different at *p* < 0.05. CU = uncoated cantaloupe at day 1, CUD5 = uncoated cantaloupe stored for 5 days, CXD5 = Xanthan coated cantaloupe stored for 5 days, CXL75 = Xanthan-*L. plantarum* LAB 75 coated cantaloupe at day 5 and CXB5 = Xanthan-*Bifidobacterium longum* coated cantaloupe fresh cuts). (**B**). Effect of coating on total chlorophyll in melons. Data is presented as means, and error bars are standard deviations from the mean. Bars for each melon cultivar, with different letters, are significantly different at *p* < 0.05. HU = uncoated cantaloupe at day 1, HUD5 = uncoated cantaloupe stored for 5 days, HXD5 = Xanthan-coated cantaloupe stored for 5 days, HXL75 = Xanthan-*Lactobacillus plantarum* LAB 75 coated cantaloupe at day 5 and HXB5 = Xanthan-*Bifidobacterium longum* coated honeydew melon fresh cuts).

Carotenoids are responsible for the orange color of cantaloupes. The green color of vegetables and fruits, including honeydew melons, is due to chlorophyll. These pigments are highly sensitive to storage temperature, packaging, coating, and duration and degrade rapidly, altering the color of fresh-cut melons [81]. During storage, the total carotenoid content of cantaloupes decreased significantly (*p* < 0.05), and 71.42% of total carotenoids were lost on the fifth day of storage in uncoated cantaloup fresh cuts (CUD5) (Figure 8A). However, the coating helped in sustaining the carotenoid content. On the other hand, cantaloup fresh cuts coated with CXL75 reduced the loss of total carotenoids by 7.0%, followed by CXB5 (−8.07%). During processing and storage, carotenoids undergo isomerization and oxidation. Contact with acids and light during storage promotes the conversion of trans-carotenoids to cis-carotenoids, thereby reducing their vitamin A activity and color [82]. Rodriguez-Amaya et al. [82] reported that foods that have higher than 0.02 mg/g of carotenoid are good sources of this pigment. However, the recommended dietary intake (DI) for different carotenoids varies. Since beta carotene is used as a standard to determine total carotenoids, its daily intake ranges from 0.15 to 2.68 mg. Abdel-Aal et al. [83] suggested that a DI of 5 to 6 mg/day could be advantageous. Consuming 100 g of CXL75 fresh cut would possibly provide 22% of carotenoids (beta carotene) to the recommended DI. Thus, consumers can consider this fresh-cut cantaloupe for its beneficial properties.

Fresh cuts of uncoated honeydew melon (HUD5) that were stored for 5 days at 5 °C experienced a significant loss of chlorophyll (45.45%) (Figure 8B). However, fresh honeydew melon cuts coated with *L. plantarum* (HXL75) showed a reduction of 8.48% in chlorophyll loss. Similarly, fresh cuts of honeydew melon coated with a probiotic coating containing *B. longum* (HXB5) showed a total chlorophyll loss of 13.63%. Xanthan-coated honeydew melon experienced a loss of 28.18% of its chlorophyll content. Therefore, the HXL75 probiotic coating was effective in protecting the chlorophyll content in fresh-cut honeydew melons. The degradation of chlorophyll is the primary cause of tissue senescence, as well as the activities of enzymes that degrade chlorophyll, such as chlorophyll oxidase and peroxidase [84]. The presence of probiotic bacteria in xanthan coatings could have prevented chlorophyll oxidation and reduced oxygen diffusion [23]. It was found that konjac gum coated with *L. plantarum* strains, including *L. plantarum* AF1, *L. plantarum* LU5, and *L. plantarum* LP3, could significantly reduce chlorophyll loss in fresh cuts of kiwi fruit stored at 4 °C for 5 days [23].

### 3.6. Probiotic Coatings and Ascorbic Acid Content

Based on Table 4, the fresh-cut cantaloupe and honeydew melons had the highest ascorbic acid (AA) content on day 0. However, uncoated cantaloupe and honeydew melon fresh cuts lost 39.52% and 44.73% of their AA content on the fifth day at 5 °C. Meanwhile, cantaloupes and honeydews coated with xanthan gum lost 30.92% and 39.47%, respectively. The probiotic coating of CXB5 reduced the loss of AA content in cantaloupe and honeydew melon by 25.57% and 15.75%, respectively. Conversely, CXL75 significantly reduced AA loss in cantaloupe and honeydew melon by 11.64% and 2.63%, respectively. The process of converting ascorbic acid into dehydroascorbic acid can happen through the direct action of the enzyme ascorbate oxidase or through the action of oxidizing enzymes like peroxidase, which occurs due to minimal processing in plant tissues. However, retaining ascorbic acid in fruits is possible using edible coatings that decrease oxygen diffusion and reduce respiration rates during storage [85]. According to Oms-Oliu, Soliva-Fortuny, and Martin-Belloso [86], fresh-cut melons coated with polysaccharide-based edible coating retained higher levels of ascorbic acid compared to samples without coatings. Probiotic bacteria may also help retain vitamins in fruits by reducing pathogen populations, decreasing oxygen diffusion, or reducing moisture loss. Additionally, kiwi fruits coated with konjac gum and *L plantarum* strains, including *L plantarum* AF1, *L plantarum* LU5, and *L plantarum* LP3, significantly retained ascorbic acid when stored for five days at 4 °C [75]. The recommended daily allowance of vitamin C for adult men is 90 mg, 75 mg for women, and 45 mg for children. This implies that consumption of 100 g of CX75 would contribute 24%, 29%, and 48.2%, and 100 g of HLX75 would contribute 24%, 28.2%, and 47% to the RDA for men, women, and children, respectively.

### 3.7. Probiotic Coatings and Total Phenols

After being stored for 5 days at 5 °C, uncoated fresh-cut cantaloupe and honeydew melons showed a reduction in TPC by 29.77% and 50.78%, respectively (Table 4). However, fresh-cut cantaloupe and honeydew melons coated with xanthan displayed a decrease in TPC by 11.68% and 23.22%, respectively, after cold storage. On the other hand, probiotic coatings CXL75 and HXL75 reduced the TPC in cantaloupe and honeydew melon fresh cuts by 12.96% and 23.51%, respectively, when compared to the uncoated day 0 samples. Similarly, CXB5 and HXB5 reduced the TPC in cantaloupe and honeydew melon fresh cuts by 14.20% and 23.21%, respectively, compared to the uncoated day 0 samples. This suggests that the probiotic cells lining the fresh-cut melon surface reduced the diffusion of oxygen and the enzymatic oxidation of polyphenols [75,87]. These results agreed with the detected lighter color of cantaloupe and honeydew melon fresh-cut samples coated with CXL75 and HXL75, respectively, probably owing to homogeneous bacterial layers adding to good visual quality. 

### 3.8. Probiotic Coatings and Antioxidant Activity

Despite the reduction in antioxidant power (FRAP) during storage in all coated and uncoated fruit compared to samples taken on day 0 (CU and HU), cantaloupe melon fresh cuts coated with probiotic coating CXL75 and honeydew melon fresh cuts coated with probiotic coatings HXB5 and HXL75 retained more antioxidant power than all other treatments (Table 4). During storage, fruit senescence damages cell structures, which exposes the phenolic compounds to oxidizing enzymes and thus reduces antioxidant activity. The higher antioxidant power observed with probiotic coating coated fresh cut melons could be the result of higher phenolic compounds, the strain’s antioxidant activity [88], the edible coating’s lower oxygen permeability, and lower decay [89]. Moreover, although the antioxidant scavenging activity DPPH and ABTS decreased during cold storage in coated and uncoated melon fresh cuts compared to day 0, the probiotic coating CXL75 retained the DPPH and ABTS activity in cantaloupe melon fresh cuts more efficiently than the HXB5 coating or other treatments. The decline in the antioxidant power during digestion can be influenced by the decrease in the TPC, possibly owing to the degradation of phenolics to other products that have a lower DPPH antioxidant capacity [90]. The ABTS behavior can also be associated with the phenolic compounds present in fruits and vegetables [90]. The decline of total carotenoids and chlorophyll can also affect these antioxidants, which can further reduce the antioxidant power present. Cantaloupe melon fresh cuts coated with probiotic coating CXL75. In contrast, probiotic-coated honeydew melon samples did not exhibit significant differences in antioxidant activity compared to xanthan gum-coated samples. A similar protective effect of *L. plantarum* was shown by Konjac-based edible coatings on fresh-cut kiwis [75]. Compared to DPPH analysis, the ABTS scavenging activity can detect lipophilic and hydrophilic antioxidants such as flavonoids, hydroxycinnamates, and carotenoids [91].

## 4. Conclusions

According to our findings, xanthan gum-based edible coatings are suitable carriers of *L. plantarum* strains onto fresh-cut cantaloupes and honeydew melons. FTIR examination supported the integration of probiotic bacteria into stable probiotic coatings with ζ-potentials ranging from −39.7 to −51.4 mV and a PdI of 1. The probiotic coatings exhibited typical pseudoplastic behavior, with viscosity curves decreasing as the shear rate increased. This study’s thermal stability analysis revealed a continuous and multi-step weight reduction, demonstrating how the edible coating’s components interact. The population of probiotics in the edible coatings was well maintained over the storage period of fresh-cut melon cubes. The probiotic treatment improved the overall acceptability of fresh-cut melon cubes and retained their antioxidant properties. The addition of *L. plantarum 75* to xanthan gum exhibited the highest preservation impact and can be recommended for the fresh-cut industry. When *L. plantarum 75* was added to the xanthan coatings, the color characteristics, pigments (carotenoids and total chlorophyll), ascorbic acid, total phenols, and antioxidant activity (FRAP, DPPH, ABTS) were significantly preserved compared to the coated and untreated control samples. The use of polysaccharides is cheaper as they are plant-based, which allows the product to be more accessible to a vast spectrum of consumers. Also, because xanthan is not composed of any dairy, the coating is suitable for vegans, vegetarians, and lactose-intolerant individuals who desire to utilize the health benefits of consuming probiotic bacteria. In order to increase the usefulness and acceptability of these films and coatings for use in a range of culinary applications, researchers and industry professionals are expected to focus on enhancing these materials’ mechanical properties, barrier capabilities, and stability. Additionally, efforts to develop edible films and coatings with enhanced antibacterial properties are likely to continue. The loading of *L. plantarum 75* in xanthan improved the functionality of the coating, and this can be useful information for the fresh-cut/probiotic bacteria industry. Probiotic coatings should be tested for bioaccessibility of antioxidant compounds and survival of embedded probiotics in the gastrointestinal tract by in vitro digestion to better understand their effectiveness. 

## Figures and Tables

**Table 1:** Zeta potential of xanthan pure xanthan gum, xanthan gum + *L. plantarum 75,* and xanthan gum + *Bifidobacterium longum* coating.

Sample Name	Z-Ave (nm)	PdI	ZP mV
Xanthan gum	420.4	1	−50.5
Xanthan + *B. longum*	498.1	1	−39.7
Xanthan + *L. plantarum 75*	641.5	1	−51.4

**Table 2:** The tensile strength of xanthan pure xanthan gum, xanthan gum + *L. plantarum 75*, and xanthan gum + *Bifidobacterium longum* coating.

**Coating Formulation**	**Thickness (mm)**	**Tensile Strength (MPa)**	**Maximum Tensile Strain (%)**
Xanthan gum	0.38 ^a^ ± 0.04	1 ^a^ ± 0.4	79.39 ^a^ ± 10.3
Xanthan gum+ *L. plantarum 75*	0.42 ^b^ ±0.05	0.74 ^b^ ± 0.2	42.0 ^c^ ± 18.9
Xanthan gum+ *B. longum*	0.42 ^b^ ± 0.04	0.83 ^c^ ± 0.4	67.1 ^b^ ± 12.4

Values are means± standard deviation. Values down the column (of the same cultivar) with different letters are significantly different at *p* < 0.05.

**Table 3:** Effect of the coating on the color properties of cantaloupe and honeydew melons after storage for 5 days.

	L*	a*	b*	C*	hº
Cantaloupe					
CU	45.60 ± 1.01 ^a^	9.72 ± 1.56 ^d^	29.00 ± 1.97 ^a^	30.59 ± 2.34 ^a^	n/a
CUD5	29.25 ± 6.80 ^d^	14.52 ± 1.74 ^a^	15.86 ± 5.91 ^d^	28.90 ± 3.20 ^bc^	n/a
CXD5	35.39 ± 1.81 ^c^	14.13 ± 0.59 ^ab^	17.82 ± 0.12 ^c^	26.23 ± 0.04 ^c^	n/a
CXL75	42.52 ± 2.60 ^b^	12.88 ± 2.87 ^c^	20.42 ± 2.94 ^b^	31.24 ± 0.30 ^a^	n/a
CXB5	39.72 ± 2.25 ^c^	14.38 ± 0.36 ^a^	17.34 ± 3.16 ^c^	25.97 ± 2.99 ^c^	n/a
LSD	1.073	2.763	2.078	7.800	
Honeydew					
HU	46.23 ± 2.95 ^a^	−2.40 ± 0.95 ^c^	11.65 ± 2.05 ^c^	20.61 ± 2.18 ^bc^	114.55 ± 1.75 ^a^
HUD5	19.57 ± 0.81 ^d^	−0.46 ± 0.21 ^b^	7.42 ± 0.63 ^d^	15.96 ± 0.64 ^d^	113.86 ± 0.53 ^a^
HX5	25.59 ± 1.12 ^c^	−9.04 ± 0.86 ^d^	19.10 ± 1.34 ^a^	21.14 ± 1.57 ^b^	115.34 ± 0.48 ^a^
HXL75	29.72 ± 2.25 ^b^	1.38 ± 0.36 ^a^	17.34 ± 3.16 ^b^	25.97 ± 2.99 ^a^	114.42 ± 0.95 ^a^
HXB5	28.57 ± 5.27 ^b^	−1.26 ± 0.51 ^b^	8.40 ± 1.26 ^d^	17.18 ± 1.35 ^c^	114.99 ± 0.72 ^a^
LSD	2.211	4.717	1.946	1.289	ns

Values are means ± standard deviation. Values down the column (of the same cultivar) with different letters are significantly different at *p* < 0.05. CU uncoated cantaloupe fresh cuts, CUD5 = uncoated cantaloupe fresh cuts, CXD5 = xanthan coated cantaloupe fresh cuts, CXL75 = Xanthan-*L. plantarum* LAB 75 coated cantaloupe fresh cuts and CXB5 = Xanthan-*Bifidobacterium longum* coated cantaloupe fresh cuts, HU = uncoated honeydew melons, HUD5 = uncoated honeydew fresh cuts, HX5 = xanthan coated honeydew fresh cuts, HXL75 = Xanthan-*L. plantarum* LAB 75 coated honeydew melons, and HXB5 = Xanthan-*Bifidobacterium longum* coated honeydew melons fresh cuts.

**Table 4:** Effect of coating on the antioxidant properties of cantaloupe and honeydew melons after storage for 5 days.

	AA (mg/100 g FW)	TPC (mg/100 g GAE FW)	FRAP (mM TE/100 g FW)	DPPH IC50 (mg/mL)	ABTS IC50 (mg/mL)
Cantaloupe					
CU	24.57 ± 1.57 ^a^	30.63 ± 0.039 ^a^	2.03 ± 0.002 ^a^	22.27 ± 0.53 ^d^	3.71 ± 0.07 ^c^
CUD5	14.86 ± 1.28 ^e^	21.51 ± 0.001 ^e^	1.44 ± 0.002 ^e^	53.73 ± 0.06 ^a^	6.80 ± 0.20 ^a^
CXB5	19.43 ± 1.28 ^c^	26.28 ± 0.006 ^d^	1.80 ± 0.001^b^	41.37 ± 0.10 ^b^	4.29 ± 0.11 ^b^
CXD5	17.71 ± 1.28 ^d^	27.05 ± 0.015 ^b^	1.61 ± 0.004 ^d^	41.32 ± 0.49 ^b^	4.40 ± 0.09 ^b^
CXL75	21.71 ± 1.57 ^b^	26.66 ± 0.016 ^c^	1.78 ± 0.001 ^c^	35.44 ± 0.39 ^c^	3.75 ± 0.0 ^c^
LSD	1.317	0.00591	0.004043	0.2389	0.0298
Honeydew					
HU	21.71 ± 1.57 ^a^	27.35 ± 0.034 ^a^	2.43 ± 0.01 ^a^	27.56 ± 0.06 ^c^	3.03 ± 0.14 ^c^
HUD5	12.00 ± 1.28 ^e^	13.46 ± 0.020 ^e^	1.40 ± 0.01 ^d^	66.26 ± 0.09 ^a^	5.35 ± 0.06 ^a^
HXB5	18.29 ± 1.57 ^c^	20.72 ± 0.002 ^d^	1.94 ± 0.00 ^b^	44.57 ± 0.00 ^b^	4.60 ± 0.002 ^b^
HXD5	13.14 ± 1.57 ^e^	21.00 ± 0.013 ^b^	1.84 ± 0.002 ^c^	44.73 ± 0.04 ^b^	4.62 ± 0.002 ^b^
HXL75	21.14 ± 1.57 ^b^	20.92 ± 0.00 ^c^	1.93 ± 0.001 ^b^	44.40 ± 0.00 ^b^	4.61 ± 0.001 ^b^
LSD	1.862	0.00836	0.005717	0.3378	0.0422

Values are means ± standard deviation. Values down the column (of the same cultivar) with different letters are significantly different at *p* < 0.05. CU = uncoated cantaloupe melon fresh cuts at day 1, CUD5 = uncoated cantaloupe melon fresh cuts stored for 5 days, CXD5 = Xanthan coated cantaloupe stored for 5 days, CXL75 = Xanthan-*L. plantarum* LAB 75 coated cantaloupe melon fresh cuts at day 5, and CXB5 = Xanthan-Bifidobacterium longum coated cantaloupe melon fresh cuts. HU = uncoated cantaloupe melon fresh cuts at day 1, HUD5 = uncoated cantaloupe melon fresh cuts, HXD5 = Xanthan coated cantaloupe melon fresh cuts HXL75 = Xanthan-*Lactobacillus plantarum* LAB 75 coated cantaloupe melon fresh cuts at day 5, and HXB5 = Xanthan-*Bifidobacterium longum* coated honeydew melon fresh cuts.

## Data Availability

The original contributions presented in the study are included in the article, further inquiries can be directed to the corresponding author.
